# Prevalence and Factors Associated with Anaemia and Undernutrition Among Children Aged 6–24 Months in Rural Tanzania

**DOI:** 10.3390/ijerph22060962

**Published:** 2025-06-19

**Authors:** Naelijwa Mshanga, Sally Moore, Neema Kassim, Carolyn I. Auma, Yun Yun Gong, Haikael D. Martin

**Affiliations:** 1 School of Life Sciences and Bioengineering, The Nelson Mandela African Institution of Science and Technology, Arusha P.O. Box 447, Tanzania; 2 School of Food Science and Nutrition, Faculty of Environment, University of Leeds, Leeds LS2 9JT, UK

**Keywords:** Tanzania, 6–24 months old, anaemia, undernutrition

## Abstract

Background: Anaemia and undernutrition remain a significant public health problem in low and middle-income countries (LMICs), particularly affecting under-five children. In Tanzania, the prevalence of anaemia and undernutrition in under-five children is still high; however, less is known about the prevalence and predictors of these conditions in rural areas. Therefore, the current cross-sectional study presents the prevalence and determinants of anaemia and undernutrition among 457 children aged 6–24 months in the Babati and Hanang districts of Tanzania. Method: Haemoglobin concentration was assessed through capillary blood samples. Anaemia was classified according to WHO 2011 guidelines using a cut-off of <11.0 g/dL for children under five, while the WHO 2006 growth indicators were used to classify the nutritional status (i.e., stunting, wasting and underweight). Results: The results from this study show that 32%, 32%, 20% and 4% of children aged 6–24 months were anaemic, stunted, underweight and wasted, respectively, while only 33% had an adequate minimum dietary diversity (MDD). In addition, the child’s age (13–24 months) was significantly associated with anaemia (AOR: 2.1 95% CI 1.4, 3.1), stunting (AOR:17.4 95% CI 10.3, 29.4) and underweight (AOR: 15.9 95% CI 7.9, 32.0). Moreover, male children were three times more likely to be wasted (AOR: 3.5 95% CI 1.1, 10.9) than their female counterparts. Anaemia and stunting were the most prevalent nutritional disorders among 6–24-month-old children in the Hanang and Babati districts. Moreover, age (13–24 months) was found to be the common predictor for anaemia, stunting and underweight. Conclusion: The observed association between age and anaemia, as well as undernutrition, suggest that age may be an essential factor when designing nutrition-related programs in similar rural settings.

## 1. Introduction

Anaemia is a disorder associated with a low count of red blood cells and haemoglobin. Haemoglobin comprises a simple protein called globin, and an iron called haem; therefore, a low iron status can lead to reduced haemoglobin levels and result in anaemia. This is supported by the World Health Organization (WHO), which reports that more than 50% of anaemia cases are caused by iron deficiency [1]. In LMICs, anaemia mainly affects women of reproductive age (15–49 years), pregnant women and children under five years [2]. Globally, anaemia cases stand at 42% in children under five [2], which is lower compared to the prevalence of anaemia (60%) in sub-Saharan Africa [3]. Undernutrition is assessed in a variety of ways; wasting is defined as low weight for length (WLZ), stunting is low length for age (LAZ), and underweight is low weight for age (WAZ), categorized as below −2 standard deviation from the median [4]. A Global Nutrition Organization highlighted that within Africa, 32% of children were stunted and 5% were wasted in East Africa; meanwhile, the total regional prevalence for stunting was 30% and that for wasting was 6% [5].

Anaemia in children under five can be caused by a low maternal iron status, the consumption of plant-based complementary foods with low bioavailable iron, malaria infection, and gastrointestinal disorders such as diarrhoea, which might affect iron absorption in the body [6,7,8]. These factors are also linked to undernutrition in children under five years. Thus, the reduced dietary intake of nutrient-rich foods, infections, and gastrointestinal disorders, i.e., diarrhoea, might lead to wasting, stunting and underweight in young children [9,10,11]. Anaemia, stunting, wasting and underweight are caused largely by the poor dietary intake of protein, energy, fat, and micronutrient-rich foods [12,13].

The WHO defines adequate minimum dietary diversity (MDD) as the consumption of five out of eight food groups on the previous day for children aged 6–24 months [14]. However, meeting the MDD has been a challenge, especially in LMICs, where most children under five consume less than five food groups in a day [15,16,17]. The reason for this might be due to the poor socio-economic status of the household, which reduces the purchasing power of nutrient-dense foods [16]. Moreover, a lack of nutrition knowledge regarding the appropriateness of complementary feeding practices among mothers and caregivers and seasonality affect the availability of certain types of foods [18,19,20]. Dietary diversity can be linked to nutritional status, as shown in a study performed in 32 Sub-Saharan African countries [16]. The study found that 6–23-month-old children who met the MDD had a 12%, 17%, and 13% reduced risk of being stunted, underweight, and wasted, respectively, compared to the ones with inadequate dietary diversity [16].

Anaemia, wasting, stunting and underweight in children can lead to a loss of appetite and diminished neural and cognitive development, which may negatively affect a child’s academic performance [21,22]. Additionally, these undernutrition disorders lead to low immunity, which increases the chances of getting diseases and infections. As a result, it affects the child’s dietary intake and nutrient metabolism [23,24,25]. Recent national data from Tanzania show that stunting stands at 30%, wasting at 3%, underweight at 12% and anaemia at 59% in children under five [17].

However, there is a scarcity of data on the nutritional status of children in rural areas like the Babati and Hanang districts since most of the data are regional and national. Therefore, this present study sought to assess the prevalence and determinants of anaemia, wasting, stunting, and underweight among 6–24-month-old children living in the Babati and Hanang districts. These districts are unique in that most residents are agro-pastoralists, i.e., graze animals and cultivate crops, leading to a distinct and unique dietary pattern. In addition, the current study is part of a bigger study on aflatoxin exposure [26] in which the Babati and Hanang districts were purposively selected as they are among the districts with high maize production in Tanzania, and maize is one of the foods that is highly susceptible to aflatoxin contamination [27,28,29].

## 2. Materials and Methods

### 2.1. Study Area, Design and Participants Recruitment

Babati and Hanang are among the six districts in the Manyara region, Tanzania. The Manyara region is in the Northern zone of Tanzania, within the latitude of 04°10′ S and longitude of 34°15′ S, with a total area of 17,190 sq m. The health centres were randomly selected to represent other centres in the respective districts.

A cross-sectional study was conducted among mother–child pairs of children aged 6–24 months from October to November 2022. Participants were recruited from the Reproductive and Child Health Clinics (RCHs), including the mobile clinics in each selected ward. Upon reaching the health facility, systematic random sampling was conducted to select the prospective participants using clinic cards. The potential participants were informed about the study and invited to join. Participants willing to participate were assessed for their eligibility, with the criteria including children aged between 6 and 24 months and children who were not sick three days before and on the interview day. The child’s sickness was confirmed by asking the mother/caregiver and the health practitioner before enrolment of the study participants.

### 2.2. Sample Size

Since the current study was part of a larger aflatoxin project, the sample size was calculated based on findings from a previous aflatoxin study conducted in Haydom Ward, Manyara region, due to the limited availability of studies from the Babati and Hanang districts.

**Figure 1:**
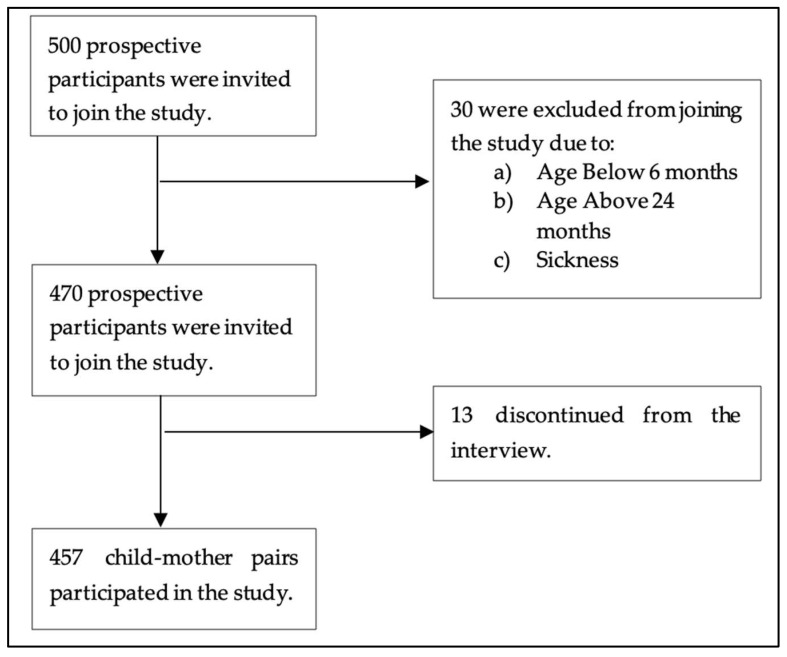
Schematic diagram showing participant recruitment and sample size.

The sample size was determined using the Kothari formula [30]: n = [(Z^2^ × P(1 − P))/d^2^]; here, P was the estimated prevalence of aflatoxin exposure (72%) among children under 36 months in Haydom, Mbulu District, Manyara region [31]. The sample size calculation used a 95% confidence level (Z = 1.96), 4% absolute precision (d = 0.04), and 80% statistical power. Based on these parameters, a sample size of 485 participants was proposed. Ultimately, 457 mother–child pairs participated in the study (Figure 1). In cases where a mother had more than one child aged 6–24 months, one child was randomly selected.

### 2.3. Assessment of Anthropometry, Anaemia and Dietary Diversity

Experienced research assistants with Human Nutrition backgrounds and two nurses from each RCH were recruited to collect anthropometric data such as the children’s length and weight, measure their haemoglobin level, and assess their dietary intake.

#### 2.3.1. Anthropometry Assessment

The length of the children was measured to the nearest 0.1 cm using a length board (210 Seca, Hamburg, Germany). Each length was taken with the child lying down, face up, on the wooden length board with five major body points touching the length board. Weight was measured to the nearest 0.1 kg and was taken in light clothing using a weighing scale (874 Seca, Hamburg, Germany). For both weight and length, measurements were taken in triplicate, and the mean of each measurement was calculated and recorded as a final measurement. The WAZ, LAZ, and WLZ growth indices were determined by using ENA for SMART software 2020 (version: Jan/11); meanwhile, the children with WAZ, LAZ and WLZ below −2 standard deviation of the median were classified as underweight, stunted, and wasted, respectively. Moreover, children between with ≤−2 SD and ≥−3 SD of the median were classified as moderately underweight, stunted, or wasted, while those with <−3 SD were classified as severely underweight, stunted, or wasted [4].

#### 2.3.2. Anaemia Diagnosis

Haemoglobin assessment was conducted in the field using a portable HemoCue^®^Hb 201+ machine (manufactured by HemoCue AB Company in Ängelholm, Sweden) which was validated before use. Two to three drops of blood were put in the microcuvette of the HemoCue^®^Hb 201+, and the haemoglobin readings were recorded in a few seconds. The measurements were performed in triplicate, and the mean of each measurement was calculated and recorded as the final measurement. The current study considered the cut-off points of a haemoglobin concentration of ≥11 g/dL, 10–10.9 g/dL, 7–9.9 g/dL and <7 g/dL as normal, mild, moderate and severe anaemia, respectively [1]. Mothers were immediately informed of their children’s haemoglobin test results. Children found to have severe anaemia were referred to paediatricians at the respective district hospitals for further evaluation and appropriate intervention, which could include blood transfusion or nutritional supplementation.

#### 2.3.3. Dietary Intake Assessment

The mothers and caregivers of children aged 6–24 months responded to a validated food frequency questionnaire (FFQ) [32]. From these, the child’s dietary diversity was calculated by assessing the intake of eight food groups in the past 24 h based on WHO guidelines [32]. The eight food groups included Group 1: Cereal, grain, roots, and tubers; Group 2: Breast milk; Group 3: Legumes and nuts; Group 4: Dairy products; Group 5: Flesh foods; Group 6: Eggs; Group 7: Vitamin A-rich fruits and vegetables; and Group 8: fruits and vegetables. For example, when a mother reported giving the child porridge made of maize flour or sorghum in the past 24 h, the consumed food was placed in group 1, or when a mother gave her child cow’s milk, this response was placed in group 4. Thereafter, adequate minimum dietary diversity was attained if a child consumed five or more groups out of the eight food groups and inadequate dietary diversity was attained if a child consumed below five food groups [14].

### 2.4. Data Analysis

The current study assumed that anaemia and undernutrition in children under five persist even in districts with high food production. To assess the factors that could have contributed to this, the dependent variables used in this study were anaemia, stunting, wasting, and underweight. The child’s age in months, gender, the number of children under the age of five the mother had, food groups, and MDD were considered as the independent variables.

Data were analyzed using Statistical Packages for Social Science (SPSS) Version 28.0. The original data were recorded in Excel and transferred to the SPSS software. Descriptive statistics were presented in terms of the frequency and percentage of the socio-demographic characteristics of the child and mother, MDD, stunting, wasting, underweight, and anaemia in children. Unadjusted and adjusted logistic regression evaluated the factors associated with stunting, underweight, wasting, and anaemia. The adjusted logistic regression model included all variables that showed a significant association in the unadjusted logistic regression analysis.

### 2.5. Ethical Considerations

This study was conducted according to the guidelines in the Declaration of Helsinki. All procedures involving human subjects were approved by the [Tanzania National Institute for Medical Research; NIMR/R.8a/Vol.IX/4077]. Written and verbal informed consent was obtained from all subjects. Verbal consent was witnessed and formally recorded.

## 3. Results

### 3.1. Socio-Demographic Characteristics

As shown in Table 1, most study participants were from the Babati District, likely due to higher birth rates in Babati compared to the Hanang District. Participants were evenly distributed by sex, with most children aged between 6 and 12 months. Most mothers were between 18 and 29 years old, married, and had completed at least primary school education. Additionally, the majority of mothers reported exclusively breastfeeding their children for the first six months and continued breastfeeding for up to two years.

### 3.2. Prevalence of Stunting, Wasting, Underweight and Anaemia Among 6–24 Months Old Children

**Figure 2:**
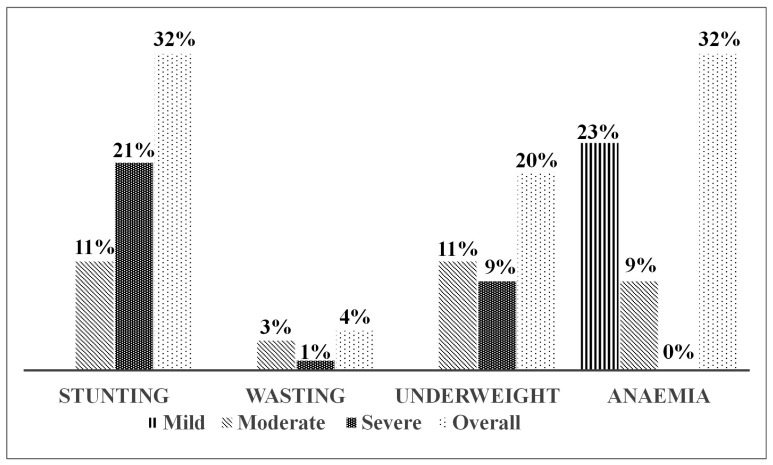
Percentage of mild, moderate and severe undernutrition and anaemia among children aged 6–24 months.

The findings from this study indicate that 32%, 4%, 20% and 32% of children aged 6–24 months were stunted, wasted, underweight and anaemic, respectively. Figure 2 presents the distribution of overall, mild, moderate and severe forms of these conditions.

### 3.3. Food Group Consumption Amongst 6–24 Months Old Children in Hanang and Babati Districts

**Figure 3:**
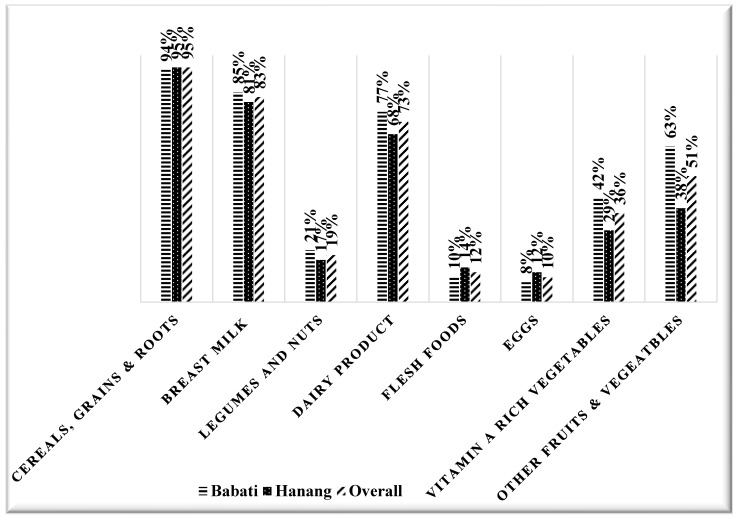
Percentage of dietary intake of different food groups among children aged 6–24 months per districts and overall.

Among the eight food groups used to calculate MDD in children, the cereal, grains, roots and tubers group was the most consumed group. In contrast, the egg group was the least consumed food group, as shown in Figure 3. Regarding fruit and vegetable intake, the Babati district consumed more fruits and vegetables and vitamin A-rich fruits and vegetables than the Hanang district, as shown in Figure 3. Meanwhile, only 33% of our study participants had attained the MDD.

### 3.4. Factors Associated with Stunting, Underweight, Wasting and Anaemia Among 6–24 Months Old Children in Babati and Hanang Districts in Unadjusted Odds Ratio

In the unadjusted logistic regression model, as shown in Table 2, factors such as a child being aged between 13 and 24 months (COR: 15.8 95% CI 9.5, 26.3) and a mother having more than three under-five children (COR: 3.4 95% CI 1.7, 6.9) increased the odds of the child being stunted. Meanwhile, the intake of cereals, grains, roots and tubers (COR: 0.3 95% CI 0.1, 0.8), legumes and nuts (COR: 0.4 95% CI 0.2, 0.8), dairy products (COR: 0.5 95% CI 0.3, 0.8) and vitamin A-rich fruits and vegetables (COR: 0.56 95% CI 0.3–0.8) reduced the odds of being stunted. Moreover, a child being aged between 13 and 24 months (COR: 15.7 95% CI 7.8, 31.5) and a mother having more than three under-five children (COR: 2.2 95% CI 1.0, 4.5) increased the odds of the child being underweight, while the intake of dairy products (COR: 0.5 95% CI 0.3, 0.8) and vitamin A-rich fruits and vegetables (COR: 0.5 95% CI 0.3, 0.89) decreased the odds of children being underweight. Being a male child (COR: 3.4 95% CI 1.1, 10.6) rather than a female child increased the odds of being wasted by three times. Children aged 13–24 months (COR: 2.2 95% CI 1.5, 3.3) were two times more likely to be anaemic compared to the younger children (6–12 months). Other factors such as the MDD, intake of fresh foods, eggs and other fruits and vegetables were not associated with stunting, underweight, wasting or anaemia.

### 3.5. Factors Associated with Stunting, Underweight, Wasting and Anaemia Among 6–24 Months Old Children in Babati and Hanang Districts in Adjusted Odds Ratio

In the adjusted logistic regression model, we found that children aged between 13 and 23 months had a higher risk of being stunted (AOR: 17.4 95% CI 10.3, 29.4), under-weight (AOR: 15.9 95% CI 7.9, 32.0) and anaemic (AOR: 2.1 95% CI 1.4, 3.1) than younger children (6–12 months). In terms of gender, a male child was three times more likely to be wasted (AOR: 3.5 95% CI 1.1, 10.9) than their female counterparts, highlighting a significant gender disparity that requires immediate attention. Moreover, the intake of cereals, grains, roots and tubers (AOR: 0.2 95% CI 0.1, 0.8), and legumes (AOR: 0.3 95% CI 0.2, 0.6) reduced the odds of being stunted. However, no other factors such as the number of children and the intake of dairy products and vitamin A-rich fruits and vegetables were found to be associated with anaemia, stunting, underweight or wasting (Table 3).

## 4. Discussion

The present study aimed to assess the prevalence and factors associated with anaemia and undernutrition among children aged 6–24 months in the Hanang and Babati districts of the Manyara region, Tanzania. In this study, we found that one-third of children were stunted and anaemic, with age in months (13–24 months) being the common predictor for anaemia, stunting and underweight.

Regarding dietary intake, cereals, grains, roots, and tubers was the most commonly consumed food group. These findings are consistent with two studies conducted in Tanzania [33,34], which reported cereals, roots, and tubers as the most consumed food group and that primarily used in the complementary foods of younger children (aged 6–24 months). Inadequate MDD was evident in most study participants (67%). These findings are consistent with those reported in the Tanzania Demographic Health Survey (TDHS-MIS), which found that most children aged 6–23 months (88%) had inadequate MDD [17]. Similarly, other studies in Tanzania found inadequate MDD among children under five [15,35]. The inadequate MDD and high intake of the cereal food group might be due to the traditional use of cereal-based complementary foods for young children in many Tanzanian communities [33,36,37,38]. In addition, the low socio-economic status of the Manyara residents may reduce their purchasing ability to buy food from other food groups (i.e., vegetables, legumes, nuts, etc.), apart from what they mainly produce as agro-pastoralists (maize and dairy foods) [15].

The prevalence of stunting (32%), wasting (4%), underweight (19%) and anaemia (32%) reported in this study aligns with the TDHS-MIS of 2022, which presents a national prevalence of 30% for stunting, 3% for wasting, 12% for underweight and 59% for anaemia among children under five years [17]. The lower prevalence of anaemia reported in this study might be attributed to seasonality, as the current study was conducted during the rainy season (November), which typically increases the availability, accessibility and affordability of vegetables [39,40]. Consequently, food prices drop with increased supply, leading to increased dietary diversification, which might have reduced the anaemia cases in the current study [15].

Interestingly, the current study found that male children had higher odds of being wasted than their female counterparts. This was consistent with other Tanzanian studies that found that female children have lower odds of being wasted than their male counterparts [17,41,42]. This can be explained by the fact that male children tend to be more playful than female children, resulting in higher energy expenditure compared to female children [9,43,44,45]. In addition, most male children tend to play outside of their home environment, which exposes them to different environmental risks and increases their chances of infections, further exacerbating the risk of wasting [42,46,47].

Stunting was more prevalent in older children (13–23 months), which is in line with other studies in Tanzania [17,41]. These results can be explained by the fact that stunting is more evident towards the end of the first 24 months in children. This signifies the repeated poor intake of food or recurrent infection at a younger age, which is evident later in life [48,49,50]. Moreover, children aged 13 to 24 months showed a higher likelihood of being underweight compared to their younger counterparts. This finding is supported by two studies conducted in Tanzania, which reported an increase in underweight cases as children grow older [17,51]. This may be attributed to increased physical activity and movement during this age, which require more energy, while mothers tend to focus their feeding practices on their younger children [42,51].

In adjusted logistic regression, the current study found that the intake of cereals grains, roots and tubers, as well as legumes and nuts, significantly decreased the odds of being stunted. These results align with a study performed in Tanzania, which found that the increased consumption of legumes, nuts, and carbohydrate-rich foods was associated with a reduced risk of stunting in children [52]. The consumption of protein-rich foods such as cow’s milk, legumes, and nuts may stimulate the production of Insulin-like Growth Factor 1 (IGF-1) [53]. When IGF-1 is stimulated, it promotes longitudinal bone growth and skeletal maturation, which can lead to an increase in height [53]. Moreover, the consumption of carbohydrate-rich foods (i.e., grains, roots and tubers) plays a crucial role in providing energy and supporting growth in young children, thereby facilitating growth and reducing stunting [54].

Another interesting finding was seen in the effect of age on anaemia; thus, older children (ages 13–24 months) were found to be more anaemic than younger children (ages 6–12 months). This aligns with findings from a study conducted in Tanzania [40]. The higher prevalence of anaemia in older children may be linked to their increased iron requirements as they grow [55]. Additionally, participants reported using complementary foods that were plant-based and contained low bioavailable iron (Fe^3+^) and phytates (iron inhibitors) [56], leading to a persistent reduction in iron intake, which becomes evident later in life.

While different categories of anaemia were assessed, no severe cases were observed. Most children presented with mild anaemia, indicating a likely chronic deficiency linked to limited sources of iron-rich or bioavailable iron, such as plant-based complementary foods. Similarly, undernutrition, particularly underweight and wasting, was primarily moderate rather than severe. However, a higher proportion of children had severe stunting compared to the other forms of undernutrition, highlighting persistent, long-term inadequacies in dietary quality rather than acute food insecurity. These findings align with the dietary patterns of agro-pastoralist communities, where consumption is primarily cereal-based and lacks diversity [57]. While this cross-sectional study does not evaluate the effectiveness of interventions, it highlights the importance of future research and programming in similar rural settings to consider context-specific approaches such as community-based nutrition education using locally available foods, seasonal diet planning, and/or food fortification. These strategies, tailored to the specific needs of each community, could significantly improve dietary quality and reduce the burden of nutritional deficiencies.

Strengths and limitations: One of the strengths of this study is its comprehensive reporting of nutritional status, including the prevalence of anaemia, stunting, underweight and wasting in children, specifically in the Babati and Hanang districts, as these results can be generalized to the respective districts. However, the current study is limited by the use of FFQ and MDD to assess dietary quality, which may have missed the quantity of food consumed, which is an essential component in evaluating dietary practices among children under five. Another limitation is the use of a cross-sectional study design, which restricted data collection to a single month and, therefore, did not account for seasonal variations in food consumption. Although the current study was well communicated to the community through local announcements and sensitization efforts, the study was conducted in RCH clinics within the hospital or dispensaries, which might have missed children who did not attend RCH clinics. Though the prevalence of malaria infection among children in the Manyara region is less than 1% [17], the current study did not directly assess infections such as malaria and worm infestations, which are known confounders of anaemia and undernutrition. To minimize potential confounding, we assessed whether children had accessed key health services within the three months before the interview. Specifically, we confirmed that participants had received deworming tablets to reduce the risk of worm infections, routine immunizations, and vitamin A supplementation. While infections were not directly measured, these preventive measures likely helped reduce their potential impact on our findings.

## 5. Conclusions

Anaemia and stunting were identified as the most prevalent forms of undernutrition among children aged 6–24 months in the Babati and Hanang districts. These conditions were significantly associated with an older age in children (13–24 months). Future nutrition programs in these areas may benefit from prioritizing strategies that directly address the determinants identified in the current study.

## Figures and Tables

**Table 1:** Socio-demographic characteristics of the study participants (mother-child dyads).

Variables	n (%)
District	
Babati	276 (60%)
Hanang	181 (40%)
Child’s Sex	
Male	235 (51%)
Female	222 (49%)
Child’s Age	
6–12 months	257 (56%)
13–24 months	200 (44%)
Mother’s Age	
<18 years	10 (2%)
18–29 years	280 (61%)
30–39 years	144 (32%)
>40 years	23 (5%)
Mother Marital Status	
Single/divorced/widowed	54 (12%)
Married	403 (88%)
Mother Education Level	
Illiterate	52 (11%)
Primary School	318 (70%)
Secondary School	75 (16%)
University/College	12 (3%)
Exclusive Breastfeeding Practice	
<than 6 months	131 (29%)
Up to 6 months	326 (71%)
Continuation of Breastfeeding up to 2 years	
Yes	384 (84%)
No	73 (16%)

**Table 2:** Factors associated with stunting, underweight, wasting and anaemia by unadjusted odds ratio.

Variables	N	Stunting			Underweight			Wasting			Anaemia		
		n (%)	COR (95% CI)	*p*-Value	n (%)	COR (95% CI)	*p*-Value	n (%)	COR (95% CI)	*p*-Value	n (%)	COR (95% CI)	*p*-Value
Districts													
Babati	276	86 (31%)	1		50 (18%)	1		12 (4%)	1		68 (25%)	1	
Hanang	181	60 (33%)	0.8 (0.4, 1.2)	0.39	38 (21%)	0.8 (0.5, 1.2)	0.26	6 (3%)	0.5 (0.2, 1.4)	0.19	77 (43%)	1.3 (0.9, 1.9)	0.19
Age (Months)													
6–12	257	26 (10%)	1		10 (4%)	1		12 (5%)	1		63 (24%)	1	
13–24	200	120 (60%)	15.9 (9.6, 26.3)	<0.001 *	78 (39%)	15.8 (7.9, 31.6)	<0.001 *	6 (3%)	0.6 (0.2, 1.7)	0.36	82 (41%)	2.1 (1.4, 3.2)	<0.001 *
Gender													
Female	222	69 (31%)	1		38 (17%)	1		4 (1.8%)	1		64 (29%)	1	
Male	235	77 (33%)	1.1 (0.7, 1.6)	0.59	50 (21%)	1.3(0.8, 2.1)	0.26	14 (6%)	3.5 (1.1, 10.7)	0.03 *	81 (34%)	1.3 (0.9, 2.0)	0.19
Number of Under-five Children a mother had													
1	236	70 (29%)	1		37 (16%)	1		6 (3%)	1		76 (32%)	1	
2	176	51 (29%)	1.01(0.6, 1.5)	0.94	38 (22%)	1.4 (0.8, 2.4)	0.12	11 (6%)	2.5 (0.9, 7.0)	0.07	57 (32%)	1.0 (0.6, 1.5)	0.96
≥3	45	25 (56%)	3.4 (1.7, 6.9)	<0.001 *	13 (29%)	2.2 (1.0, 4.5)	0.03 *	1 (2%)	0.8 (0.1, 7.4)	0.90	12 (27%)	0.7 (0.4, 1.5)	0.76
MDD													
Adequate	152	41 (30%)	1		25 (16%)	1		5 (3%)	1		49 (32%)	1	
Inadequate	305	105 (34%)	1.4 (0.9, 2.2)	0.12	63 (21%)	1.3 (0.8, 2.2)	0.28	13 (4%)	1.3 (0.5, 3.7)	0.62	96 (31%)	0.9 (0.6, 1.5)	0.87
Consumption of Food Group in Preceding 24 h													
Cereals, grains, roots and tubers													
No	24	13 (54%)	1		6 (25%)	1		2 (8%)	1		9 (38%)	1	
Yes	433	133 (31%)	0.4 (0.2, 0.9)	0.02 *	82 (20%)	0.7 (0.3, 1.8)	0.47	16 (4%)	0.4 (0.09, 1.9)	0.27	136 (31%)	0.8 (0.3, 1.8)	0.53
Legumes and Nuts													
No	367	128 (35%)	1		77 (21%)	1		16 (4%)	1		121 (33%)	1	
Yes	90	18 (20%)	0.5 (0.3, 0.8)	0.008 *	11 (12%)	0.5 (0.3,1.03)	0.06	2 (2%)	0.5 (0.1, 2.2)	0.35	24 (27%)	0.7(0.4, 1.2)	0.25
Dairy Products													
No	119	50 (42%)	1		32 (27%)	1		2 (2%)	1		48 (40%)	1	
Yes	338	96 (28%)	0.5 (0.3, 0.8)	0.003 *	56 (17%)	0.54 (0.3, 0.9)	0.015 *	16 (5%)	2.9 (0.7, 12.8)	0.16	97 (29%)	0.5 (0.4, 1.2)	0.08
Flesh Foods													
No	403	127 (32%)	1		82 (20%)	1		15 (4%)	1		123 (31%)	1	
Yes	54	19 (35%)	1.2 (0.7, 2.2)	0.52	6 (11%)	0.5 (0.2, 1.2)	0.11	3 (5%)	1.5 (0.4, 5.4)	0.51	22 (41%)	1.5 (0.9, 2.8)	0.13
Eggs													
No	412	133 (32%)	1		80 (19%)	1		18 (4%)	1		130 (32%)	1	
Yes	45	13 (29%)	0.8 (0.4, 1.5)	0.51	8 (18%)	0.9 (0.4, 2.0)	0.79	0 (0%)	0.4 (0.06–1.7)	0.17	15 (33%)	1.1 (0.6, 2.1)	0.80
Vitamin A-Rich Fruits and Vegetables													
No	287	104 (36%)	1		65 (23%)	1		10 (3%)	1		94 (33%)	1	1
Yes	170	42 (25%)	0.6 (0.4, 0.9)	0.01 *	23 (14%)	0.5 (0.3, 0.9)	0.018 *	8 (5%)	1.4 (0.5, 3.5)	0.52	51 (30%)	0.9 (0.6, 1.3)	0.54
Other Fruits and Vegetables													
No	213	70 (33%)	1		47 (22%)	1		10 (4%)	1		72 (34%)	1	1
Yes	244	76 (31%)	0.9 (0.6, 1.4)	0.66	41 (17%)	0.7 (0.4, 1.1)	0.15	8 (3%)	0.7 (0.3, 1.7)	0.44	73 (30%)	0.8 (0.6, 1.2)	0.37

Note: COR: Crude/Unadjusted Odds Ratio, PV: *p* Value, * *p* < 0.05.

**Table 3:** Factors associated with stunting, underweight, wasting and anaemia by adjusted odds ratio.

Variable	Stunting AOR (95% CI)	*p*-Value	Underweight AOR (95% CI)	*p*-Value	Wasting AOR (95% CI)	*p*-Value	Anaemia AOR (95% CI)	*p*-Value
Age (Months)								
6–12	1		1		1		1	
13–24	17.4 (10.3, 29.4)	<0.001 *	15.9 (7.9, 32.0)	<0.001 *	0.58 (0.2, 1.6)	0.30	2.1 (1.4, 3.1)	0.001 *
Gender								
Female	1		1		1		1	
Male	1.1 (0.6, 1.9)	0.57	1.3 (0.8, 2.2)	0.32	3.5 (1.1, 10.9)	0.03 *	1.3 (0.9, 2.0)	0.21
Number of Under-five Children in a Household								
1	1		1		1		1	
2	0.9 (0.5, 1.5)	0.71	1.3 (0.7, 2.3)	0.29	2.7 (0.9, 7.7)	0.05	0.9 (0.6, 1.4)	0.84
≥3	2.0 (0.8, 4.6)	0.09	1.1 (0.5, 2.6)	0.65	1.04 (0.1, 9.2)	0.97	0.5 (0.2, 1.2)	0.15
Consumption of Food Group in Preceding 24 h								
Cereals, Grains, Roots and Tubers								
No	1		1		1		1	
Yes	0.2 (0.1, 0.8)	0.02 *	0.8 (0.3, 2.3)	0.65	0.4 (0.08, 2.0)	0.28	0.8 (0.3, 1.8)	0.56
Legumes and Nuts								
No	1		1		1		1	
Yes	0.3 (0.2, 0.6)	0.001 *	0.5 (0.2, 1.0)	0.07	0.6 (0.1, 2.5)	0.46	0.7 (0.4, 1.3)	0.29
Dairy Products								
No	1		1		1		1	
Yes	0.8 (0.5, 1.4)	0.48	0.9 (0.5, 1.76)	0.97	0.4 (0.1, 1.8)	0.23	1.4 (0.9, 2.3)	0.11
Vitamin A-rich Fruits and Vegetables								
No	1		1		1		1	
Yes	1.2 (0.7, 2.2)	0.51	1.3 (0.07, 2.5)	0.35	0.5 (0.02, 1.5)	0.21	0.9 (0.6, 1.5)	0.72

Note: AOR: Adjusted Odds Ratio, PV: *p* Value, * *p* < 0.05.

## Data Availability

The raw data supporting the conclusions of this article will be made available by the authors on request.

## Abbreviations

The following abbreviations are used in this manuscript:

HB	Haemoglobin
IGF-1	Insulin-like growth factor 1
LAZ	Length for Age
MDD	Minimum Dietary Diversity
RCH	Reproductive Child Health
SPSS	Statistical Package for Social Science
WAZ	Weight for Age
WLZ	Weight for Length
WHO	World Health Organization
